# Universal Testing Policy for COVID-19 in Pregnancy: A Systematic Review

**DOI:** 10.3389/fpubh.2022.588269

**Published:** 2022-02-08

**Authors:** Nur Amirah Farhanah Hashim, Zaleha Abdullah Mahdy, Rahana Abdul Rahman, Aida Hani Mohd Kalok, Rosnah Sutan

**Affiliations:** ^1^Department of Obstetrics and Gynaecology, Faculty of Medicine, Universiti Kebangsaan Malaysia Medical Centre, Cheras, Malaysia; ^2^Department of Community Health, Faculty of Medicine, Universiti Kebangsaan Malaysia Medical Centre, Cheras, Malaysia

**Keywords:** severe acute respiratory syndrome coronavirus 2, pregnancy, universal testing, prevalence, policy

## Abstract

**Background:**

The coronavirus disease (COVID-19) has spread at an accelerated rate. WHO reported that in the general population, the majority are either asymptomatic or mildly infected. In view of the high risk of SARS-CoV-2 transmission from a pregnant woman to her newborn, healthcare workers and other patients, it is a raised concern whether universal testing should be implemented in this targeted population. The current guidelines have not recommended a universal testing policy. In certain European countries, however, the policy was implemented by some hospitals in regions with high prevalence of COVID-19 infection.

**Aim(s):**

To assess the justification for universal screening of pregnant women for COVID-19 prior to admission in labor through systematic review of antenatal prevalence of asymptomatic infection, hence risk of inadvertent spread of infection.

**Materials and Methods:**

Three databases confined to PubMed, Ovid and Science Direct were used to search for articles from November 2019 onwards published in the English language. The search was conducted using the keywords “COVID-19” or “coronavirus” or “SARS-CoV-2” and “pregnancy” or “pregnant” or “obstetric” or “labor” and “universal” or “testing” or “prevalence”. The review was registered with PROSPERO.

**Results:**

The search result retrieved 34 studies, with the majority consisting of retrospective cohort studies, while other studies such as prospective cohort study, research letters and a case series were also identified. A total of 19,958 pregnant women were universally tested until the date of report. Overall, the prevalence of universal testing among pregnant women presenting to labor and delivery units are higher in Western regions. From the total number of pregnant women 5.3% tested positive and among these, the majority (75.5%) did not manifest any symptoms at the time of testing.

**Conclusion:**

In areas with high prevalence of COVID-19 infection, the implementation of a universal testing policy among pregnant women presenting to labor and admission units may be cost effective in helping to curb disease transmission.

**Systematic Trial Registration:**

https://www.crd.york.ac.uk/prospero/display_record.php?ID=CRD42020184248, PROSPERO: CRD42020184248.

## Introduction

Coronavirus disease (COVID-19), a respiratory illness that is caused by a novel coronavirus (SARS-CoV-2) is a global public health crisis and emergency. Since the World Health Organization (WHO) announced it as a pandemic on 11 March 2020, the virus has continued to spread rapidly and tremendously worldwide (1). As of 6 February 2021, the number of individuals infected globally has reached over 105 million of the population, with more than 2 million deaths^1^. Large studies of five vaccine candidates' efficacy and safety results have been publicly reported through press releases and several countries have begun implementing public vaccination, however it is still too early to perceive widespread benefit^2^, as viral mutations continue to be reported.

A report by the WHO stated that approximately 80% of the COVID-19 infected population are either asymptomatic or mildly symptomatic (2). Asymptomatic patients are those who have positive test result for SARS-CoV-2 without symptom manifestation. A study by researchers at Johns Hopkins University showed that universal testing increased COVID-19 case detection by more than 200 percent in general as compared to targeted testing, and concluded that more testing resources are needed to curb the infection (3). The study also summarized that unrecognized asymptomatic cases can hinder preventive strategies, as well as increase the risk of the virus spreading (3). As pregnant women are also affected by coronavirus, this disease has drawn attention around the world, whether a universal testing policy should be imposed on all pregnant women who attend labor and admission units. In the United States, the National Institutes of Health categorized the disease severity into: asymptomatic, mild, moderate, severe critical illness (4). Knowledge regarding the capability of the virus to spread from an asymptomatic patient is still limited and poorly understood (5). Liu et al. described two out of 15 cases of pregnant women who were asymptomatic at presentation and underwent testing in view of contact history, in whom pneumonic lesion of COVID-19 was identified upon computed tomography evaluation (6). A study in an affiliated pair of New York City hospitals revealed 14 out of 43 (32.6%) pregnant women who were initially either asymptomatic and presented for obstetrically indicated labor induction, or remained asymptomatic upon presentation, and were subsequently identified to have positive COVID-19 infection upon universal testing at labor unit admission (7). Such asymptomatic pregnant women are at higher risk of infecting their newborns upon birth, healthcare workers and other patients if they are not identified.

According to the WHO, the decision to perform a test should be based on clinical and epidemiological factors that meet the suspected case definition for COVID-19 (8). In order for a test to be used for screening procedures in early disease detection, it should fulfill certain criteria such as validity, reliability, yield, cost, acceptance and follow-up services (9). It is more desirable and cost-effective to conduct universal testing in a population where the prevalence is high (10). This review therefore aims to look at reported prevalence rates of COVID-19 and thus explore the need for a universal testing policy for COVID-19 among pregnant women especially at the time of admission for delivery. It should be borne in mind that inadvertent exposure of healthcare workers to undiagnosed COVID-19 positive patients is an occupational hazard that comes with dire consequences, not only on the health and life of the worker, but also on healthcare services as a result of staff shortage due to quarantine and illness. Infection from an asymptomatic pregnant woman with COVID-19 infection who comes in labor in particular is a hazard to the healthcare worker that we should be seriously concerned about.

## Method

### Study Design

This is a systematic review of literature that was conducted in accordance with the Preferred Reporting Items for Systematic Reviews and Meta-Analyses (PRISMA) guidelines. The study protocol and review were registered with PROSPERO (CRD42020184248). Two other systematic reviews were registered simultaneously in the same PROSPERO proposal but are dealt with separately.

### Literature Search Strategy

A thorough and comprehensive literature search for studies published from November 2019 onwards was conducted and limited to English language publications. Three different electronic databases (PubMed, Ovid and Science Direct) were searched using the keywords “universal testing,” “COVID-19” and “pregnancy.” The PICOS terms used are as shown in Table 1. Additional relevant studies found from the references were also retrieved. The Boolean operator ‘AND' was used to combine parts of the subject terms and ‘OR' was used to expand the search. Only the latest publication would be chosen when there were similar studies with more than one publication.

**Table 1:** PICOS criteria for inclusion and exclusion of studies.

**Parameter**	**Inclusion criteria**	**Data extraction**
Population	Pregnant women presented to labor and delivery admission unit	Location
Intervention	Universal testing on all pregnant women presented to labor and delivery admission unit	Prevalence of positive test for COVID-19
Comparator	None	
Outcome	Pregnant women with positive test for COVID-19	Prevalence of symptomatic and asymptomatic women with COVID-19 positive test
Study	Case reports/observational studies	Type of study design

### Screening of Articles for Eligibility and Quality Assessment

The articles identified from the databases and additional resources were screened for eligibility. First, the title and abstract were screened. Second, eligible studies had to meet all the inclusion criteria developed from the research question using PICOS (Population, Intervention, Comparator, Outcome, Study) design as shown in Table 2. Exclusion criteria includes patients known to have previously been tested positive for SARS-CoV-2 infection. Full articles were retrieved and read in the event of any doubt or uncertainty regarding the content relevance during the abstract screening. After a comprehensive list of abstracts was obtained, the articles were retrieved and reviewed in full-text. One researcher screened all studies and the results were collated and reviewed by the second researcher. In the event of disagreement involving the study selection, a third reviewer would be consulted to reach a consensus.

**Table 2:** Summary of studies reviewing the outcome of universal testing and the prevalence of asymptomatic pregnant women with positive SARS-CoV-2.

**No**	**First Author**	**Country**	**Title**	**Universally tested pregnant women *N***	**COVID-19 Infection**				**Study design**	**RoB**
					**Negative *n* (%)**	**Positive**				
						**Total *n* (%)**	**Asymptomatic *n* (%)**	**Symptomatic *n* (%)**		
1	Prabhu et al. (11)	United States (New York)	Pregnancy and postpartum outcomes in a universally tested population for SARS-CoV-2 in New York City: a prospective cohort study	675	605 (89.6)	70 (10.4)	55 (78.6)	15 (21.4)	Prospective cohort	++
2	Fassett et al. (12)	United States (Los Angeles)	Universal SARS-CoV-2 screening in women admitted for delivery in a large managed care organization	3,923	3,906 (99.6)	17 (0.4)	17 (100.0)	0 (0.0)	Retrospective cohort	+
3	Vintzileos et al. (13)	United States (New York)	Screening all pregnant women admitted to labor and delivery for the virus responsible for coronavirus disease 2019	161	129 (81.1)	32 (19.9)	21 (65.6)	11 (34.4)	Retrospective cohort	++
4	Campbell et al. (14)	United States (New York)	Prevalence of SARS-CoV-2 among patients admitted for childbirth in Southern Connecticut	770	740 (96.1)	30 (3.9)	22 (73.3)	8 (26.7)	Retrospective cohort	+
5	LaCourse et al. (15)	United States (Washington)	Low prevalence of SARS-CoV-2 among pregnant and postpartum patients with universal screening in Seattle, Washington	188^a^	182 (97.3)	5 (2.7)	1 (20.0)	4 (80.0)	Retrospective cohort	++
6	Miller et al. (16)	United States (Chicago)	Clinical implications of universal severe acute respiratory syndrome coronavirus 2 (SARS-CoV-2) testing in pregnancy	635	612 (96.4)	23 (3.6)	10 (43.5)	13 (56.5)	Research letter	++
7	London et al. (17)	United States (New York)	The relationship between status at presentation and outcomes among pregnant women with COVID-19	75	65 (86.7)	10 (13.3)	10 (100.0)	0 (0.0)	Retrospective cohort	++
8	Goldfarb et al. (18)	United States (Boston)	Universal SARS-CoV-2 testing on admission to the labor and delivery unit: low prevalence among asymptomatic obstetric patients	757	737 (97.4)	20 (2.6)	9 (45.0)	11 (55.0)	Retrospective cohort	+
9	Ochiai et al. (19)	Japan	Universal screening for SARS-CoV-2 in asymptomatic obstetric patients in Tokyo, Japan	52	49 (94.2)	3 (5.8)	3 (100.0)	0 (0.0)	Retrospective cohort	++
10	Bianco et al. (20)	United States (New York)	Testing of patients and support persons for coronavirus disease 2019 (COVID-19) infection before scheduled deliveries	155	131 (84.5)	24 (15.5)	24 (100.0)	0 (0.0)	Retrospective cohort	++
11	Ferrazzi et al. (21)	Italy	SARS-CoV-2 infection testing at delivery: a clinical and epidemiological priority	1,566	1,517 (96.9)	49 (3.1)	27 (55.1)	22 (44.9)	Retrospective cohort	+
12	Herraiz et al. (22)	Spain (Madrid)	Universal screening for SARS-CoV-2 before labor admission during COVID-19 pandemic in Madrid	203^a^	199 (99.0)	2 (1.0)	1 (50.0)	1 (50.0)	Retrospective cohort	++
13	Sutton et al. (23)	United States (New York)	Universal screening for SARS-CoV-2 in women admitted for delivery	215^a^	181 (84.7)	33 (15.3)	29 (87.9)	4 (12.1)	Research letter	+++
14	Gagliardi et al. (24)	Italy (Tuscany and Liguria)	Universal severe acute respiratory syndrome coronavirus 2 testing of pregnant women admitted for delivery in 2 Italian regions	533	530 (99.4)	3 (0.6)	2 (66.7)	1 (33.3)	Research letter	++
15	Yassa et al. (25)	Turkey	Outcomes of universal SARS-CoV-2 testing program in pregnant women admitted to hospital and the adjuvant role of lung ultrasound in screening: a prospective cohort study	296	273 (92.2)	23 (7.8)	12 (52.2)	11 (47.8)	Retrospective cohort	++
16	Berkowitz et al. (26)	United States (Ohio)	Implementation of universal testing for SARS-CoV-2 in pregnant women with intended admission for delivery	518^b^	482 (98.1)	10 (1.9)	7 (70.0)	3 (30.0)	Research letter	+++
17	Santos et al. (27)	Portugal	Prevalence of SARS-CoV-2 infection in asymptomatic pregnant women and their partners in a tertiary care hospital in Portugal	428	426 (99.5)	2 (0.5)	2 (100.0)	0 (0.0)	Research letter	+++
18	Abeysuriya et al. (28)	United Kingdom	Universal screening for SARS-CoV-2 in pregnant women at term admitted to an East London maternity unit	180^c^	171 (96.1)	7 (3.9)	6 (85.7)	1 (14.3)	Retrospective cohort	++
19	Buckley et al. (29)	United States (New York)	Universal testing of patients and their support persons for severe acute respiratory syndrome coronavirus 2 when presenting for admission to labor and delivery at Mount Sinai Health System	307	257 (83.7)	50 (16.3)	50 (100.0)	0 (0.0)	Research letter	+++
20	Doria et al. (30)	Portugal	COVID-19 during pregnancy: a case series from an universally tested population from the north of Portugal	103	91 (88.4)	12 (11.6)	11 (91.7)	1 (8.3)	Case series	+++
21	Bender et al. (31)	United States (Pennsylvania)	Universal testing for severe acute respiratory syndrome coronavirus 2 in 2 Philadelphia hospitals: carrier prevalence and symptom development over 2 weeks	318	310 (97.5)	8 (2.5)	8 (100.0)	0 (0.0)	Retrospective cohort	+++
22	Vinuela et al. (32) (2020)	Spain (Madrid)	SARS-CoV-2 screening of asymptomatic women admitted for delivery must be performed with a combination of microbiological techniques: an observational study	100	91 (91.0)	9 (9.0)	9 (100.0)	0 (0.0)	Retrospective cohort	+++
23	Tanacan et al. (33)	Turkey (Ankara)	The rate of SARS-CoV-2 positivity in asymptomatic pregnant women admitted to hospital for delivery: experience of a pandemic center in Turkey	206	203 (98.5)	3 (1.5)	3 (100.0)	0 (0.0)	Prospective cohort	+++
24	Saviron-Cornudella et al. (34)	Spain (Madrid)	Severe acute respiratory syndrome coronavirus 2 (SARS-CoV-2) universal screening in gravids during labor and delivery	266	260 (97.7)	6 (2.3)	4 (66.7)	2 (33.3)	Retrospective cohort	++
25	Reale et al. (35)	United States (Massachusetts)	Patient characteristics associated with SARS-CoV-2 infection in parturients admitted for labor and delivery in Massachusetts during the spring 2020 surge: a prospective cohort study	2,945	2,852 (96.8)	93 (3.2)	80 (86.0)	13 (14.0)	Prospective cohort	+
26	Pineles et al. (36)	United States (Texas)	Racial-ethnic disparities and pregnancy outcomes in SARS-CoV-2 infection in a universally-tested cohort in Houston, Texas	935	858 (91.8)	77 (8.2)	66 (85.7)	11 (14.3)	Retrospective cohort	++
27	Naqvi et al. (37)	United States (Los Angeles)	Severe acute respiratory syndrome coronavirus 2 (SARS-CoV-2) universal testing experience on a Los Angeles labor and delivery unit	82	81 (98.8)	1 (1.2)	0l (0.0)	1 (100.0)	Prospective cohort	++
28	Mei-Dan et al. (38)	Canada (Toronto)	Questionnaire-based vs universal PCR testing for SARS-CoV-2 in women admitted for delivery	446	442 (99.1)	4 (0.9)	3 (66.7)	1 (33.3)	Prospective cohort	+
29	Maru et al. (39)	United States (New York)	Universal screening for SARS-CoV-2 infection among pregnant women at Elmhurst Hospital Center, Queens, New York	124	78 (62.9)	46 (37.1)	33 (71.7)	13 (28.3)	Retrospective cohort	++
30	Hcini et al. (40)	French Guiana	Maternal, fetal and neonatal outcomes of large series of SARS-CoV-2 positive pregnancies in peripartum period: a single-center prospective comparative study	507	370 (73.0)	137 (27.0)	103 (75.2)	34 (24.8)	Prospective cohort	+
31	Figueiredo et al. (41)	Portugal (Porto)	Systematic screening for SARS-CoV-2 in pregnant women admitted for delivery in a Portuguese maternity	184	173 (99.9)	11 (0.1)	9 (81.8)	2 (18.2)	Prospective cohort	+
32	Waghmare et al. (42)	India (Maharashtra)	Universal screening identifies asymptomatic carriers of SARS-CoV-2 among pregnant women in India	1,140	999 (87.6)	141 (12.4)	284 (73.8)	37 (26.2)	Prospective cohort	++
33	Diaz-Corvillon et al. (43)	Chile	Routine screening for SARS CoV-2 in unselected pregnant women at delivery	583	546 (93.7)	37 (6.3)	16 (43.2)	21 (56.8)	Prospective cohort	+
34	Blitz et al. (44)	United States (New York)	Universal testing for coronavirus 2019 in pregnant women admitted for delivery: prevalence of peripartum infection and rate of asymptomatic carriers at four New York hospitals within an integrated healthcare system	382	318 (83.3)	64 (16.7)	45 (70.3)	19 (29.7)	Retrospective cohort	++
	TOTAL			19,958	18,896 (94.7)	1,062 (5.3)	802 (75.5)	260 (24.5)		

*N, Pregnant women who were universally tested; a, Asymptomatic and inconclusive (n = 1); b, Results were not obtained within clinically relevant time frame (n=26); c, Two women (n = 2) excluded as one was previously suspected and other one declined testing; RoB, Risk of bias; +, Low risk of bias; ++, Medium risk of bias; +++, Serious risk of bias*.

### Data Extraction

The following information was manually extracted from each study: year and country of publication, name of first author, study design, sample size/number of pregnant women who participated, trimester, number of pregnant women with positive or negative COVID-19 infection and number of asymptomatic infected pregnant women. The relevant data extracted was organized into tables using an Excel® spreadsheet. Gray literature was searched for any written policy of universal testing for COVID-19 in pregnancy.

### Data Synthesis and Quality Assessment

Information retrieved was analyzed and interpreted. The primary outcomes assessed were the number of population with positive COVID-19 infection through universal testing, the number of asymptomatic pregnant women, and their prevalence. The information was synthesized using a narrative (descriptive) method. The quality of each study was independently evaluated by the first researcher using the Mixed Methods Appraisal Tools (45).

## Results

**Figure 1:**
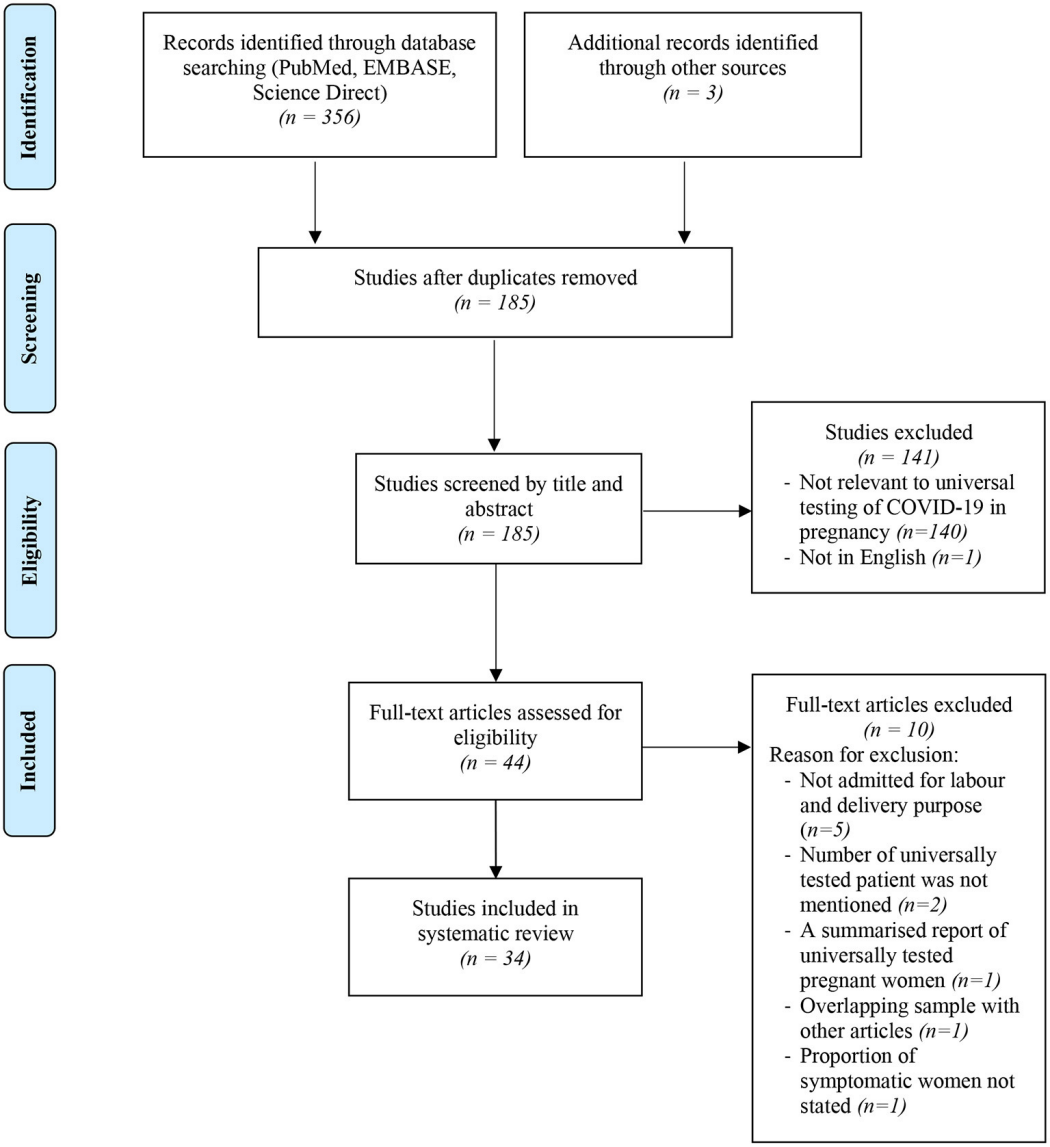
Flowchart showing inclusion in systematic review of studies reporting on prevalence of universal testing policy for COVID-19 in pregnant women.

The selection process of articles and inclusion in the systematic review was summarized in Figure 1 using the PRISMA flow diagram for systematic review. The initial search yielded a total of 356 articles. Other sources such as references from searched articles yielded three additional articles for this review. After removing the duplicates, 185 articles were screened for keywords relevance from the title and abstract. The full-text versions of the publications were reviewed in case of uncertainty. Only those that fulfill the inclusion criteria shown in Table 1 and English publications were included for eligibility assessment. The full texts of these studies were fully examined. Eventually, only a total of 34 articles were included in this review, consisting mainly of retrospective cohort studies, followed by research letter, prospective cohort studies and case series. The data from these 34 studies was further summarized in Table 2. A total of 19,958 pregnant women worldwide were universally tested for COVID-19 infection upon arrival at labor and delivery admission units.

### Risk of Bias

By using the Mixed Methods Appraisal Tools (MMAT) (3), the risk of bias of the studies were summarized in Table 2. In general, the individual studies had low to moderate range of risk of bias due to adequate approach to the research question and findings, with presence of coherence among the sources, data collection and analysis. In contrast, research letters and case series had moderate to serious risk of bias due to poor inclusion criteria. However, the clinical cases were presented clearly with clear messages provided.

### Main Findings

This systematic review reports the prevalence of universal testing policy worldwide. It is notably found that the policy is adopted mostly in Western countries, as the implementation of the policy is highest in regions such as New York, Italy, Spain and Portugal. About two thirds (13,165/19,958 or 66.0%) of the population tested were from the United States, one of the countries with the highest number of population affected by the disease. From the total number of 19,958 pregnant women tested, an average of 5.3% were found to be infected. The total positive test rate ranged from 0.4 (12) to 27.0% (40). We also found that 1.3% (260/19,958) of the total number of pregnant women presenting to labor and admission units were asymptomatic women who tested positive for COVID-19 infection. Out of the total number of positive tests for COVID-19, the proportion of asymptomatic pregnant women (75.5%) was markedly higher than symptomatic pregnant women (24.5%). Guidelines vary in terms of recommendation for testing for COVID-19 among pregnant women. The ACOG recommends universal testing in areas with high prevalence of the infection (46).The guidelines issued by the Indian Council of Medical Research recommends universal testing of “all pregnant women in/near labor who are hospitalized for delivery” (47). However, other guidelines may not state such a stand clearly (48), and may rely on clinical screening as first line (49).

## Discussion

This systematic review reports with great concern that the prevalence of asymptomatic COVID-19 patients is threefold that of symptomatic patients, thus seriously raising the question of universal testing, particularly in pregnancy, where there is prolonged close contact with multiple healthcare workers especially when the patient is in labor. Although prevalence varies across the globe and several vaccines have been successfully tested and now implemented, mutants of SARS-CoV-2 appear every so often, hence other preventive measures such as limiting contact and physical distancing still matters.

It is good to note some move toward advocating universal testing of pregnant women attending labor and delivery units, given the recent spike in the prevalence of asymptomatic COVID-19 cases worldwide. Recently, the American College of Obstetricians and Gynecologists published an update on the recommendation to consider universal testing for pregnant women especially in high prevalence areas (46). In a study by Bianco et al., universal screening using the telephone as a screening tool is inadequate as 24 patients who were previously not identified as likely to be COVID-19 positive via such screening, were tested positive from the universal testing (20). Therefore, the findings of this systematic review implies that healthcare workers and other patients are at significant risk of exposure and getting infected with COVID-19, if universal testing of pregnant women is not implemented in high prevalence areas. The alternative measure is universal precaution i.e., wearing of personal protective equipment (PPE) when handling all cases. However, universal usage of PPE will result in wastage of a precious commodity that has been in short supply.

Routine SARS-CoV-2 testing would require the use PPE. On the other hand, in the case of patients with reported symptoms but received negative results, PPE use could be avoided. In general, universal testing may result in an overall increase in terms of PPE usage. Therefore, given the potential increased need for supply, the implementation of universal testing could pose a challenge to the current hospital supply systems. An increased demand for PPE would occur and facilities with limited access to PPE would suffer greatly.

The implementation of universal testing in pregnancy, however, can act as a multipronged approach to reduce the risk of SARS-CoV-2 transmission, particularly in healthcare facilities in regions with high prevalence of the infection. In view of longer exposure between pregnant women and healthcare professionals before, during and after delivery, universal testing in this specific population can assist in infection control operations. It can help protect the safety of newborns, hospital staff, and other patients. In addition, it also allows priority clinical care to be given to both the infected mother and her baby at the time of birth and during the postpartum period, in terms of appropriate further treatment such as management of delivery, counseling for breastfeeding and newborn skin-to-skin contact. It is important to bear in mind that the COVID-19 prevalence rate is extremely fluid and has a tendency to escalate rapidly^2^, hence policies and guidelines should be formulated in a flexible manner so as to be enable prompt response with day to day changes in the situation.

Attempts have also been made to elucidate clinical or simple laboratory predictive risk factors for COVID-19 infection among pregnant women in order to proceed to conduct targeted antigen testing (50–52). This may be more feasible options in the long term, especially from the health economics point of view. It can also be implemented irrespective of the local prevalence of the disease.

The prevalence rate from universal testing appears to mirror the rate within the local general population (37). The current compiled review is useful in planning preventive strategies in the interest of the health of pregnant mothers, their babies, and mitigating the risk of healthcare workers. In regions with low COVID-19 prevalence, the approach may be different. The research question on universal testing needs to be addressed carefully. The incubation period for COVID-19 is reported to be between 5 and 14 days and the duration of immunity is still being studied. For populations with low prevalence, using a screening checklist and restricting diagnostic testing only for those with positive screening may be a more cost-effective option. A cost-effectiveness study on universal testing is in order before universal testing can be recommended as a policy. The issue of timing of testing in relation to pregnancy and labor also needs to be considered and are not easy decision points.

### Strength and Limitations

The strength of our study is as a systematic review that looks at universal testing policy for COVID-19 in pregnancy at the point of admission for labor and delivery. It is useful in guiding policy making in relation to preventive measures and testing for the infection. One limitation of this review is the nature of the studies retrieved. Although the majority of studies included are retrospective cohort studies, case reports and research letters that were retrieved are expected to have high risk of bias. Apart from that, we did not report the prevalence of COVID-19 in the general population in individual studies as they were likely to be underreported to various degrees, depending on the extent of mass testing in a particular population. As a result, we were not able to compare the prevalence of infected cases by different regions and countries. It is quite difficult to do this retrospectively for all locations as the local prevalence changes fairly rapidly and the studies were time-sensitive, several of them limiting the study period to 1 or 2 weeks only.

## Conclusion

This review looks at the outcome of a universal testing policy in terms of prevalence of asymptomatic pregnant women in various populations. Given the high rate of asymptomatic pregnant women in certain regions of the world, universal testing may provide enhanced safety to the public and healthcare workers in these areas, but cost will be increased from various angles. Although the current trend of universal testing predominates only in developed countries, more studies involving developing and less developed countries should be conducted to provide valuable information of the need for such a policy of universal testing for COVID-19 in pregnancy. Universal testing provides benefits in areas with high prevalence of disease, hence testing for the background prevalence in representative samples of pregnant women in various regions should be considered in order to guide policy making. Above all, in areas with high prevalence of COVID-19, the strategy of universal testing of pregnant women before admitting them for delivery is essential and must be implemented rigorously in order to protect the women, their newborns, and in-contact healthcare workers so as to curb the spread of infection in the community.

## Author Contributions

NAFH led the data synthesis, collection and analysis, while being supervised by ZAM. RS gave an expert clinical advice on methodology and community health, while AHMK and RAR gave expert clinical advice on obstetrics. All authors critically revised the manuscript for important intellectual content and reviewed and approved the final version.

## Conflict of Interest

The authors declare that the research was conducted in the absence of any commercial or financial relationships that could be construed as a potential conflict of interest.

## Publisher's Note

All claims expressed in this article are solely those of the authors and do not necessarily represent those of their affiliated organizations, or those of the publisher, the editors and the reviewers. Any product that may be evaluated in this article, or claim that may be made by its manufacturer, is not guaranteed or endorsed by the publisher.

## Footnotes

^1^https://www.worldometers.info/coronavirus/ (accessed February 6, 2021).

^2^https://www.who.int/news-room/q-a-detail/coronavirus-disease-(covid-19)-vaccines (accessed February 5, 2021).

## Abbreviations

COVID-19: Coronavirus Disease 2019
SARS-CoV-2: Severe Acute Respiratory Syndrome Coronavirus 2
PPE: Personal Protective Equipment
WHO: World Health Organization
CDC: Centers for Disease Control and Prevention
MMAT: Mixed Method Appraisal Tools.
